# Can Machine Learning Reduce Unnecessary Surgeries? A Retrospective Analysis Using Threshold Optimization to Prevent Negative Appendectomies in Adults

**DOI:** 10.1002/ags3.70225

**Published:** 2026-04-28

**Authors:** Ivan Males, Marko Kumric, Zvonimir Boban, Josip Vrdoljak, Dario Pecenkovic, Matej Ivanda, Marko Grahovac, Zenon Pogorelic, Josko Bozic

**Affiliations:** ^1^ Department of Surgery, Division of Abdominal Surgery University Hospital of Split Split Croatia; ^2^ Laboratory for Artificial Intelligence and Data Science in Biomedicine University of Split School of Medicine Split Croatia; ^3^ Department of Pathophysiology University of Split School of Medicine Split Croatia; ^4^ Laboratory for Cardiometabolic Research University of Split School of Medicine Split Croatia; ^5^ Department of Internal Medicine, Division of Endocrinology, Diabetology and Metabolism University Hospital of Split Split Croatia; ^6^ Department of Surgery, School of Medicine University of Split Split Croatia; ^7^ Department of Paediatric Surgery University Hospital of Split Split Croatia

**Keywords:** adult, appendectomy, appendicitis, clinical decision support systems, machine learning

## Abstract

**Aim:**

To develop and validate machine learning models using routinely available clinical and laboratory data in adults with highly suspected acute appendicitis and to assess their potential to reduce negative appendectomies.

**Methods:**

A retrospective study was conducted including adult patients who underwent appendectomy for suspected acute appendicitis between January 2020 and June 2024. Histopathology was the reference standard. Logistic regression, random forest, balanced random forest, and gradient boosting models were developed. Decision thresholds were calibrated to achieve predefined sensitivity targets. Performance was assessed using the area under the receiver operating characteristic curve (AUC) and standard diagnostic metrics. Stability was evaluated using bootstrap resampling and explainability by SHAP analysis.

**Results:**

A total of 623 patients were included, of whom 66 had a negative appendectomy. For appendicitis detection, logistic regression demonstrated the best performance with a mean AUC of 0.765. At the highest sensitivity thresholds, specificity remained low but identified a small subset of patients at minimal risk of appendicitis. For complicated appendicitis prediction, random forest performed the best with a mean AUC of 0.785. SHAP analysis identified inflammatory laboratory markers as the most influential predictors. Bootstrap analysis confirmed model stability.

**Conclusion:**

Machine learning models based on clinical and laboratory variables may provide adjunctive decision support in surgically selected adults with suspected acute appendicitis and may help identify a small subgroup in whom immediate surgery could be reconsidered at carefully selected sensitivity thresholds. Prospective external validation using a similar study design is required before clinical implementation.

## Graphical abstract

Threshold‐optimized machine learning models using routine clinical and laboratory data in 623 adults undergoing appendectomy. Logistic regression (AUC = 0.765) and random forest (AUC = 0.785) were the best‐performing models for appendicitis detection and complicated appendicitis prediction, respectively. Inflammatory markers were the most influential predictors. Models are intended as adjunctive decision support, not a replacement for standard diagnostic pathways.

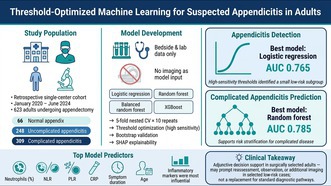

## 1 Introduction

Acute appendicitis remains one of the most common surgical emergencies worldwide, with a global age‐standardized incidence of 214 per 100 000, equalling to approximately 17 million new cases annually [1]. Despite its frequency and the widespread availability of modern imaging modalities, accurate diagnosis of acute appendicitis continues to pose significant clinical challenges [2]. The diagnostic uncertainty stems from the substantial overlap between appendicitis and numerous mimicking conditions of gastrointestinal, urologic, or gynecologic origin, particularly in female patients of reproductive age [3]. This diagnostic ambiguity results in negative appendectomies in approximately 13% of cases, with large variations between individual studies [4]. Negative appendectomy carries implications beyond unnecessary surgical intervention, with measurable risk to patients and a morbidity rate of 10%–12%. Some of the complications include surgical site infection, intra‐abdominal abscesses, and postoperative adhesions. Moreover, a mortality rate of around 1% has been reported [5]. Negative appendectomy has been linked to increased mortality in both the short and long term, even after accounting for recognized confounding factors. This persistent excess risk may reflect the presence of unrecognized underlying morbidity and underscores the importance of improving preoperative diagnostic accuracy in order to minimize avoidable surgical harm [6].

In this context, artificial intelligence and machine learning (ML) have emerged as promising tools to augment clinical decision‐making across multiple medical disciplines [7]. In the context of appendicitis diagnosis, recent studies demonstrate that ML algorithms can achieve diagnostic accuracy comparable to or exceeding experienced emergency department physicians [8]. Moreover, ML models integrating clinical, laboratory, or imaging parameters have shown superior performance compared to clinical scoring systems such as the Alvarado score, AIR, or PAS [8, 9, 10]. Furthermore, the current literature is characterized by substantial methodological heterogeneity, with different ML models developed and evaluated in different patient populations and using diverse outcome definitions and performance metrics, thereby limiting generalizability and comparability of findings [11]. When integrating such models into clinical practice, model thresholds must be tailored to reflect the relative importance of sensitivity versus specificity in a given clinical context [9].

These advances suggest that ML‐based clinical decision support systems could meaningfully improve appendicitis management by enhancing diagnostic precision, optimizing surgical decision‐making, and ultimately reducing unnecessary procedures. The aim of this study was not to develop a general diagnostic tool for patients with undifferentiated abdominal pain, but to evaluate whether ML models based on routinely available clinical and laboratory data could support decision making in adult patients with histopathologically confirmed diagnosis with high suspicion of appendicitis and to assess their potential to reduce negative appendectomy rates.

## 2 Materials and Methods

### 2.1 Study Design and Ethical Considerations

The study sample consisted of adult patients who underwent surgery under the clinical suspicion of acute appendicitis at the University Hospital of Split and were hospitalized at the Department of Surgery. Data were collected retrospectively from the electronic medical records for the period between January 2020 and June 2024. Initially, by analysing electronic medical records, 1547 entries were identified. All patients who underwent open or laparoscopic appendectomy were included, while incidental appendectomies were excluded. Patients without available pathohistological reports or with diagnoses other than acute appendicitis or a histologically normal appendix were excluded. No additional exclusion criteria were applied. The database was reviewed for data entry errors and implausible values, which were corrected or excluded, and variable formats were standardized. Histopathology findings were individually reviewed and classified as normal appendix, uncomplicated appendicitis, or complicated appendicitis. Variables with more than 30% missing data were excluded. Patients missing more than two key laboratory predictors (leukocyte count, neutrophil percentage, platelet count, C‐reactive protein, or sodium concentration) were excluded, resulting in a final dataset of 623 patients for model development.

The study was approved by the Ethics Committee of the University Hospital of Split (Approval number: 500–03/23–01/169; approval date: July 21, 2023) and conducted in accordance with the ethical principles of the Declaration of Helsinki. All personal data were anonymized prior to analysis.

### 2.2 Model Development and Validation

Four ML models were developed and compared: logistic regression (LR), random forest (RF), balanced random forest (BRF), and eXtreme gradient boosting (XGBoost). For model training and validation, a nested cross‐validation approach was applied, comprising five‐fold inner and outer cross‐validation, repeated ten times. The dataset was divided into five outer folds, with four folds used for training and one held out for testing in each iteration. Within every outer training set, an additional five‐fold cross‐validation was performed to optimize hyperparameters and calibrate decision thresholds. The optimized parameters and thresholds obtained from the inner validation were then applied to the corresponding outer test set, ensuring that model tuning and calibration were carried out independently from the test data and that performance estimates remained unbiased. Stratification was performed on the target variable due to the imbalanced dataset, ensuring representation of all target classes in each split.

Missing values were imputed using the Bagged Trees algorithm implemented via the *step_impute_bag* function from the *recipes* package in R. To prevent data leakage, imputation for validation and test sets was based solely on estimates derived from their respective training sets. The following hyperparameters were optimized during model training: for LR, the regularization penalty and mixture parameters; for the RF model, the number of variables considered at each split (mtry), the minimum node size (min_n), and the number of trees; and for the XGBoost model, the learning rate, loss reduction (gamma), number of trees, subsample ratio, tree depth, minimum node size, number of variables per split, and the number of stopping iterations. The number of trees for BRF was set explicitly to 2000.

Threshold shifting was performed to identify decision thresholds that maximized specificity while achieving predefined minimum sensitivity targets. Within each outer fold of the nested cross‐validation, thresholds were derived exclusively on inner cross‐validation predictions. Predicted appendicitis probabilities from all five inner validation folds were pooled to form a single out‐of‐fold validation set, from which a ROC curve was constructed. For each of six predefined target sensitivities (0.95, 0.96, 0.97, 0.98, 0.99, 0.995), the threshold with the highest specificity while maintaining at least the target sensitivity was identified on this pooled ROC curve. These six thresholds were then fixed and applied without modification to the corresponding outer test set to obtain unbiased estimates of sensitivity, specificity, and related performance metrics. Model performance was summarized by averaging results across 50 outer test sets (five outer folds repeated ten times) and reporting mean values with standard errors. The final best‐performing model was selected as the one with the highest mean area under the ROC curve (AUC) across these outer test evaluations as they are insensitive to threshold shifting.

Bootstrap analysis was conducted to estimate variability in model performance when retrained on different subsamples of the same population, thereby evaluating model stability against sampling fluctuations.

Modelling was performed using R (version 2025.05.0, R Core Team, Vienna, Austria). The following libraries were used: *caret*, *glmnet*, *fastshap*, *pROC*, *ROCR*, *reshape2*, *dplyr*, *tidyr*, *purrr*, *ggplot2*, *plotly*, *tidymodels*, *ranger*, *xgboost*, and *fastshap*. A global random seed was set to ensure reproducibility of all results in cases where stochastic functions were involved.

### 2.3 Model Explainability

The SHAP (SHapley Additive exPlanations) framework was used to clarify how clinical and laboratory features affected the model's predictions and to improve understanding of its decision‐making process. By estimating each feature's relative impact on prediction outcomes, SHAP analysis revealed which factors most influenced model behavior. This method enhances interpretability, promotes transparency, and supports the potential use of the model in clinical settings. All analyses were performed in R using the fastshap package.

### 2.4 Statistical Analysis

The normality of data distributions was assessed using the Kolmogorov–Smirnov test. Kruskal–Wallis test was used for comparisons between the groups for numerical data, while chi‐squared and Fisher's exact test were used for categorical values. A *p*‐value < 0.05 was considered statistically significant. All statistical analyses and visualizations were performed in the R programming language.

## 3 Results

In total, 623 adult patients were included in model training and evaluation, of whom 66 had a normal appendix, 248 had uncomplicated appendicitis, and 309 had complicated appendicitis. Patient characteristics are shown in Table 1.

**TABLE 1:** Patients' baseline characteristics.

Feature	Normal appendix (*n* = 66)	Uncomplicated appendicitis (*n* = 248)	Complicated appendicitis (*n* = 309)	*p*
Age, years	33 (24)	35 (22)	45 (26)	**< 0.001** *
Sex, *n* (%)				
Male	23 (34.8%)	149 (60.1%)	166 (53.7%)	**0.001** **
Female	43 (65.2%)	99 (39.9%)	143 (46.3%)	
Duration of symptoms, hours	48 (42)	20 (12)	36 (24)	**< 0.001** *
Fever, *n* (%)				
Yes	21 (32.8%)	43 (17.9%)	112 (38.2%)	**< 0.001** **
No	43 (67.2%)	97 (82.1%)	181 (61.8%)	
Nausea, *n* (%)				
Yes	29 (43.9%)	112 (46.3%)	167 (56%)	0.039**
No	37 (56.1%)	130 (53.7%)	131 (44%)	
Vomiting, *n* (%)				
Yes	12 (18.2%)	77 (31.4%)	124 (41.1%)	**< 0.001** **
No	54 (81.8%)	168 (68.6%)	178 (58.9%)	
Rebound tenderness, *n* (%)				
Yes	51 (77.3%)	183 (75%)	252 (82.1%)	0.123**
No	15 (22.7%)	61 (25%)	55 (17.9%)	
Pain migration, *n* (%)				
Yes	25 (38.5%)	124 (50.2%)	140 (45.8%)	0.212**
No	40 (61.5%)	103 (49.8%)	166 (54.2%)	
LRQ pain, *n* (%)				
Yes	62 (93.9%)	238 (96.4%)	285 (92.2%)	**0.124** **
No	4 (6.1%)	9 (3.6%)	24 (7.8%)	
WBC, 10^9^/L	10.3 (5.1)	13,1 (5.65)	14.6 (6.15)	**< 0.001** *
Neutrophil, %	75.15 (15.8)	80.55 (11.4)	83,9 (9.98)	**< 0.001** *
Lymphocyte, %	15.15 (12.8)	12.1 (9.1)	9.4 (6.95)	**< 0.001** *
MPV, fL	9,45 (1.7)	9.6 (1.5)	9.5 (1.5)	**0.494** *
NLR	4.95 (4.47)	6.83 (6.42)	9,38 (8.89)	**< 0.001** *
PLR	153.12 (103.26)	148.97 (94.75)	175.45 (116.89)	**< 0.001** *
CRP, mg/L	36.15 (66.3)	21.4 (44.18)	83.95 (127.75)	**< 0.001** *
Sodium, mmol/L	140 (3)	139 (3)	138 (4)	**< 0.001** *

*Note:* Data are presented as median (IQR) or *n* (%). Bold values indicate statistically significant results, defined as *p* < 0.05.

Abbreviations: LRQ, lower right quadrant; MPV, mean platelet value; NLR, neutrophil to lymphocyte ratio; PLR, platelet to lymphocyte ratio; WBC, white blood count.

* Kruskal–Wallis test.

** Chi squared test.

The proportion of missing values ranged from 0% to 5.3% across all variables. Sodium levels had the highest percentage of missing data (5.3%), followed by body temperature (4.2%). Other variables with smaller amounts of missing data included nausea, vomiting, symptom duration, rebound tenderness, pain migration, CRP, and LRQ pain. All remaining variables were complete, with no missing values (Table 2).

**TABLE 2:** Percentage of missing values in the dataset.

Feature	Percentage of missing values
Sodium concentration	5.3
Fever	4.2
Nausea	2.7
Vomiting	1.6
Length of symptoms	1.4
Rebound tenderness	0.9
Pain migration	0.8
CRP	0.3
LRQ pain	0.1
Sex	0
Age	0
WBC	0
MPV	0
Neutrophil percentage	0
Lymphocyte percentage	0
PLR	0
NLR	0

Abbreviations: LRQ, lower right quadrant; MPV, mean platelet volume; NLR, neutrophil to lymphocyte ratio; PLR, platelet to lymphocyte ratio; WBC, white blood count.

### 3.1 Appendicitis Detection Model

**FIGURE 1:**
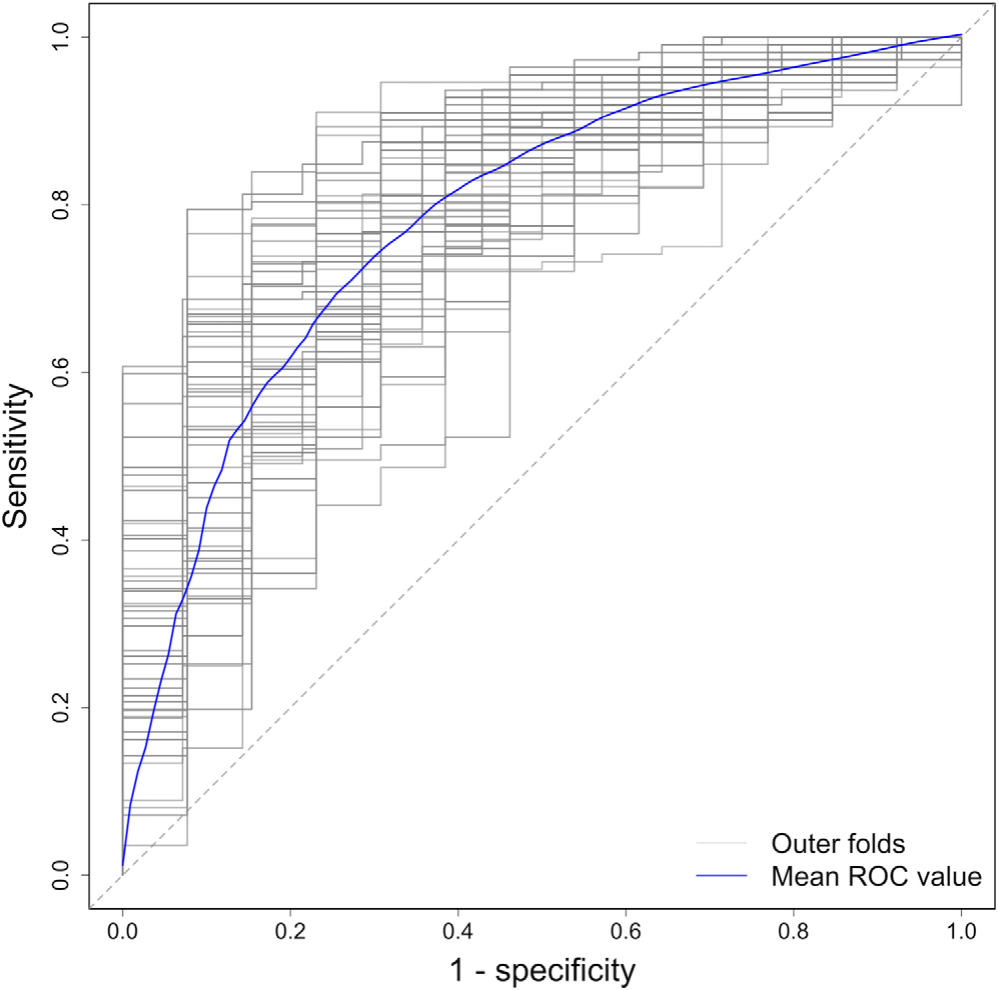
ROC curve of the average logistic regression model and ROC curves for each outer fold. Blue curve represents the mean ROC curve across repeated test folds, and the grey curves represent ROC curves from individual outer folds. The ROC curves describe overall model discrimination across decision thresholds; predefined minimum sensitivity targets were subsequently used during inner cross‐validation to select operating thresholds that maximized specificity.

Four ML models—LR, RF, BRF, and XGBoost—were evaluated with a primary focus on maximizing sensitivity for detecting acute appendicitis. To achieve this, decision thresholds were adjusted to enhance model sensitivity. The best overall performance was achieved with the LR model, which reached an AUC of 0.765 ± 0.001 (Figure 1). Furthermore, multiple threshold‐adjusted models were generated to ensure target sensitivities ranging from 0.95 to 0.995 (Table 3). At the highest achieved sensitivity of 0.998, the corresponding specificity was 0.038 for LR. Overall, sensitivities ranged from 0.944 to 0.998 and specificities ranged from 0.26 to 0.022 depending on the targeted sensitivity.

**TABLE 3:** Models' performance in acute appendicitis detection.

Model and thresholds	AUC	Sensitivity	Specificity	Accuracy	PPV	NPV	F1
RF							
Threshold 1	0.745 ± 0.011	0.952 ± 0.003	0.259 ± 0.016	0.880 ± 0.003	0.918 ± 0.002	0.398 ± 0.023	0.935 ± 0.002
Threshold 2		0.962 ± 0.003	0.195 ± 0.014	0.883 ± 0.003	0.912 ± 0.001	0.387 ± 0.027	0.936 ± 0.001
Threshold 3		0.97 ± 0.002	0.165 ± 0.014	0.886 ± 0.002	0.910 ± 0.001	0.405 ± 0.033	0.939 ± 0.001
Threshold 4		0.982 ± 0.002	0.100 ± 0.011	0.891 ± 0.002	0.905 ± 0.001	0.413 ± 0.048	0.943 ± 0.001
Threshold 5		0.994 ± 0.001	0.049 ± 0.008	0.896 ± 0.001	0.901 ± 0.001	0.488 ± 0.071	0.945 ± 0.001
Threshold 6		0.997 ± 0.001	0.022 ± 0.006	0.896 ± 0.001	0.898 ± 0.001	0.441 ± 0.101	0.945 ± 0.001
BRF							
Threshold 1	0.757 ± 0.01	0.95 ± 0.004	0.236 ± 0.017	0.874 ± 0.003	0.913 ± 0.002	0.378 ± 0.023	0.931 ± 0.002
Threshold 2		0.962 ± 0.003	0.203 ± 0.017	0.881 ± 0.003	0.911 ± 0.002	0.401 ± 0.028	0.935 ± 0.001
Threshold 3		0.97 ± 0.003	0.155 ± 0.014	0.883 ± 0.003	0.907 ± 0.001	0.405 ± 0.033	0.937 ± 0.001
Threshold 4		0.981 ± 0.002	0.097 ± 0.011	0.887 ± 0.002	0.902 ± 0.001	0.401 ± 0.048	0.94 ± 0.001
Threshold 5		0.989 ± 0.002	0.059 ± 0.009	0.891 ± 0.001	0.899 ± 0.001	0.429 ± 0.052	0.942 ± 0.001
Threshold 6		0.994 ± 0.001	0.041 ± 0.009	0.893 ± 0.001	0.897 ± 0.001	0.431 ± 0.071	0.943 ± 0.001
XGBoost							
Threshold 1	0.723 ± 0.013	0.944 ± 0.005	0.260 ± 0.018	0.873 ± 0.004	0.918 ± 0.002	0.374 ± 0.026	0.930 ± 0.003
Threshold 2		0.952 ± 0.005	0.237 ± 0.018	0.878 ± 0.004	0.916 ± 0.002	0.377 ± 0.026	0.933 ± 0.002
Threshold 3		0.962 ± 0.005	0.200 ± 0.018	0.883 ± 0.003	0.913 ± 0.002	0.396 ± 0.025	0.936 ± 0.002
Threshold 4		0.972 ± 0.004	0.146 ± 0.018	0.887 ± 0.003	0.908 ± 0.002	0.407 ± 0.042	0.939 ± 0.002
Threshold 5		0.984 ± 0.004	0.094 ± 0.017	0.892 ± 0.003	0.904 ± 0.001	0.459 ± 0.061	0.942 ± 0.002
Threshold 6		0.989 ± 0.004	0.062 ± 0.014	0.893 ± 0.002	0.902 ± 0.001	0.533 ± 0.081	0.943 ± 0.002
LR							
Threshold 1	**0.765 ± 0.001**	0.954 ± 0.003	0.228 ± 0.019	0.879 ± 0.003	0.915 ± 0.002	0.365 ± 0.022	0.934 ± 0.002
Threshold 2		0.965 ± 0.003	0.178 ± 0.016	0.884 ± 0.003	0.911 ± 0.002	0.389 ± 0.029	0.937 ± 0.001
Threshold 3		0.975 ± 0.003	0.134 ± 0.015	0.888 ± 0.002	0.907 ± 0.001	0.405 ± 0.04	0.940 ± 0.001
Threshold 4		0.982 ± 0.002	0.102 ± 0.013	0.891 ± 0.002	0.905 ± 0.001	0.43 ± 0.051	0.942 ± 0.001
Threshold 5		0.993 ± 0.002	0.07 ± 0.011	0.898 ± 0.002	0.903 ± 0.001	0.61 ± 0.07	0.946 ± 0.001
Threshold 6		0.998 ± 0.001	0.038 ± 0.009	0.899 ± 0.001	0.900 ± 0.001	0.722 ± 0.094	0.946 ± 0.001

*Note:* Thresholds 1–6—thresholds with minimum sensitivity of 0.95, 0.96, 0.97, 0.98, 0.99 and 0.995. Values are presented as mean ± standard error.

Abbreviations: AUC, area under the curve; BRF, balanced random forest; F1, F1 score; LR, logistic regression; NPV, negative predictive value; PPV, positive predictive value; RF, random forest; XGB, eXtreme Gradient Boosting. Bold values indicate statistically significant results, defined as *p* < 0.05.

To further evaluate the performance of the developed models for all thresholds, bootstrap analysis was conducted using 2000 iterations with resampled datasets from the original population. In each iteration, the created model was retrained on the bootstrap sample and tested on the corresponding out‐of‐bag instances, allowing assessment of variability in performance when the model is applied to different subsamples of the same population. This procedure estimated model stability in relation to sampling fluctuations. Performance metrics for all evaluated models with their thresholds are presented in Table A1.

**FIGURE 2:**
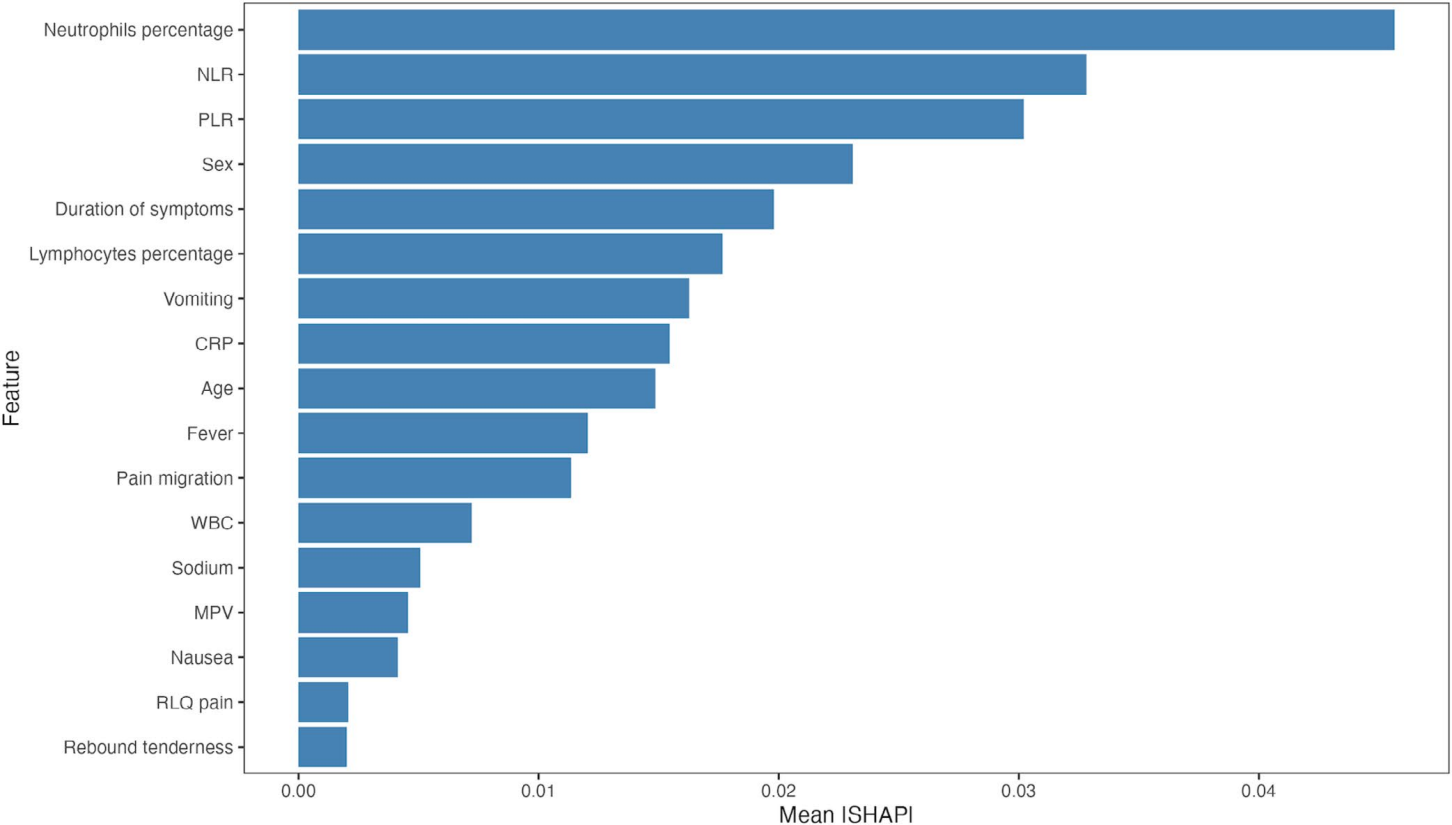
Feature importance of logistic regression model for appendicitis prediction for the threshold with the highest minimum sensitivity criteria based on Shapley values, shown as average of the absolute value for each fold. Larger values indicate greater overall contribution of that feature to model predictions. LRQ, Lower right quadrant; MPV, Mean platelet volume; NLR, Neutrophil to lymphocyte ratio; PLR, Platelet to lymphocyte ratio.

To better understand how the LR model processed the information and what it has deemed as important features, Shapley values were calculated with 10 000 permutations for each outer fold and then averaged. For the threshold with the highest minimum sensitivity criteria, the five most important values were neutrophil percentage, NLR, PLR, sex, and lymphocyte percentage (Figure 2).

When SHAP values were examined by prediction outcome for the same threshold, distinct feature importance rankings were observed across true positives, false positives, true negatives, and false negatives. For both true positive and false positive predictions, the highest mean absolute SHAP values were associated with neutrophil percentage, NLR, PLR, sex, and symptom duration, in that order. In true negative predictions, symptom duration exhibited the highest SHAP contribution, followed by neutrophil percentage, NLR, lymphocyte percentage, and PLR. For false negative predictions, neutrophil percentage showed the greatest SHAP impact, followed by symptom duration, NLR, lymphocyte percentage, and PLR.

### 3.2 Model for Prediction of Complicated Appendicitis

**FIGURE 3:**
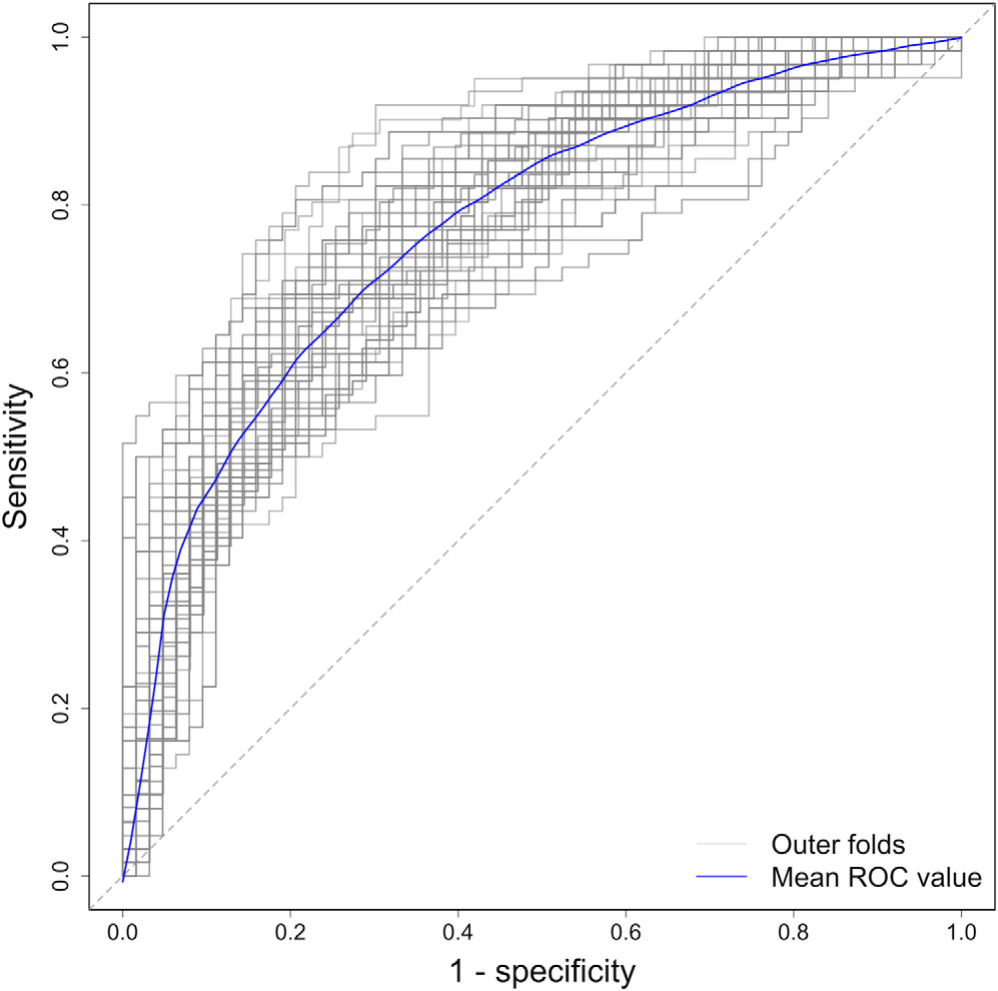
ROC curve of the average random forest model and ROC curves for each outer fold. Blue curve represents the mean ROC curve across repeated test folds, and the grey curves represent ROC curves from individual outer folds. The ROC curves describe overall model discrimination across decision thresholds; predefined minimum sensitivity targets were subsequently used during inner cross‐validation to select operating thresholds that maximized specificity.

Using the same methodology as in the previous analysis, patients with uncomplicated appendicitis were grouped together with those with a normal appendix and compared with patients with complicated appendicitis, reflecting a potential role for initial non‐operative management. Out of the developed models, RF showed the best results with an AUC of 0.785 ± 0.006 (Figure 3). At the highest achieved sensitivity of 0.997, the corresponding specificity was 0.057 (Table 4). Overall, sensitivities ranged from 0.939 to 0.997 and specificities ranged from 0.320 to 0.053 depending on the targeted sensitivity.

**TABLE 4:** Models' performance in complicated appendicitis detection.

Model and thresholds	AUC	Sensitivity	Specificity	Accuracy	PPV	NPV	F1
RF							
Threshold 1	**0.785 ± 0.006**	0.946 ± 0.005	0.302 ± 0.009	0.630 ± 0.004	0.585 ± 0.003	0.854 ± 0.012	0.723 ± 0.003
Threshold 2		0.955 ± 0.005	0.254 ± 0.008	0.611 ± 0.004	0.571 ± 0.002	0.856 ± 0.013	0.714 ± 0.002
Threshold 3		0.966 ± 0.004	0.199 ± 0.009	0.589 ± 0.004	0.556 ± 0.003	0.858 ± 0.014	0.706 ± 0.002
Threshold 4		0.975 ± 0.003	0.151 ± 0.009	0.571 ± 0.004	0.544 ± 0.003	0.866 ± 0.015	0.698 ± 0.002
Threshold 5		0.988 ± 0.002	0.091 ± 0.008	0.548 ± 0.004	0.530 ± 0.002	0.904 ± 0.017	0.690 ± 0.002
Threshold 6		0.997 ± 0.001	0.057 ± 0.008	0.533 ± 0.003	0.522 ± 0.002	0.876 ± 0.030	0.684 ± 0.002
XGBoost							
Threshold 1	0.781 ± 0.005	0.939 ± 0.006	0.320 ± 0.009	0.635 ± 0.004	0.590 ± 0.003	0.844 ± 0.012	0.724 ± 0.003
Threshold 2		0.953 ± 0.005	0.276 ± 0.009	0.621 ± 0.004	0.578 ± 0.003	0.861 ± 0.013	0.719 ± 0.003
Threshold 3		0.962 ± 0.004	0.238 ± 0.009	0.607 ± 0.004	0.568 ± 0.003	0.868 ± 0.014	0.714 ± 0.003
Threshold 4		0.972 ± 0.003	0.205 ± 0.009	0.596 ± 0.004	0.560 ± 0.003	0.890 ± 0.014	0.710 ± 0.002
Threshold 5		0.983 ± 0.002	0.141 ± 0.008	0.570 ± 0.004	0.543 ± 0.002	0.903 ± 0.014	0.699 ± 0.002
Threshold 6		0.988 ± 0.004	0.108 ± 0.008	0.566 ± 0.004	0.535 ± 0.002	0.921 ± 0.014	0.694 ± 0.002
LR							
Threshold 1	0.776 ± 0.006	0.947 ± 0.006	0.235 ± 0.010	0.598 ± 0.004	0.563 ± 0.003	0.825 ± 0.015	0.706 ± 0.003
Threshold 2		0.961 ± 0.005	0.176 ± 0.010	0.576 ± 0.005	0.548 ± 0.003	0.823 ± 0.019	0.698 ± 0.003
Threshold 3		0.970 ± 0.004	0.137 ± 0.010	0.561 ± 0.004	0.539 ± 0.003	0.829 ± 0.021	0.692 ± 0.002
Threshold 4		0.98 ± 0.003	0.108 ± 0.008	0.552 ± 0.004	0.533 ± 0.002	0.859 ± 0.024	0.691 ± 0.002
Threshold 5		0.99 ± 0.002	0.074 ± 0.006	0.540 ± 0.003	0.526 ± 0.002	0.899 ± 0.025	0.687 ± 0.002
Threshold 6		0.994 ± 0.002	0.053 ± 0.005	0.532 ± 0.002	0.522 ± 0.001	0.912 ± 0.024	0.684 ± 0.001
BRF							
Threshold 1	0.769 ± 0.006	0.951 ± 0.005	0.296 ± 0.007	0.621 ± 0.003	0.571 ± 0.002	0.869 ± 0.01	0.713 ± 0.002
Threshold 2		0.961 ± 0.005	0.247 ± 0.007	0.601 ± 0.003	0.557 ± 0.002	0.877 ± 0.012	0.705 ± 0.002
Threshold 3		0.968 ± 0.004	0.215 ± 0.007	0.589 ± 0.003	0.549 ± 0.002	0.886 ± 0.011	0.700 ± 0.002
Threshold 4		0.982 ± 0.003	0.143 ± 0.008	0.559 ± 0.004	0.530 ± 0.002	0.899 ± 0.015	0.688 ± 0.002
Threshold 5		0.991 ± 0.002	0.084 ± 0.007	0.534 ± 0.003	0.516 ± 0.002	0.917 ± 0.016	0.679 ± 0.001
Threshold 6		0.994 ± 0.002	0.056 ± 0.006	0.509 ± 0.003	0.509 ± 0.002	0.911 ± 0.028	0.673 ± 0.001

*Note:* Thresholds 1–6—thresholds with minimum sensitivity of 0.95, 0.96, 0.97, 0.98, 0.99 and 0.995. Values are presented as mean ± standard error.

Abbreviations: AUC, area under the curve; BRF, balanced random forest; F1, F1 score; LR, logistic regression; NPV, negative predictive value; PPV, positive predictive value; RF, random forest; XGB, eXtreme Gradient Boosting. Bold values indicate statistically significant results, defined as *p* < 0.05.

Again, to further evaluate the performance of the developed models for all thresholds, bootstrap analysis was conducted using 2000 iterations with resampled datasets from the original population. Performance metrics for all evaluated models with their thresholds are presented in Table A2.

**FIGURE 4:**
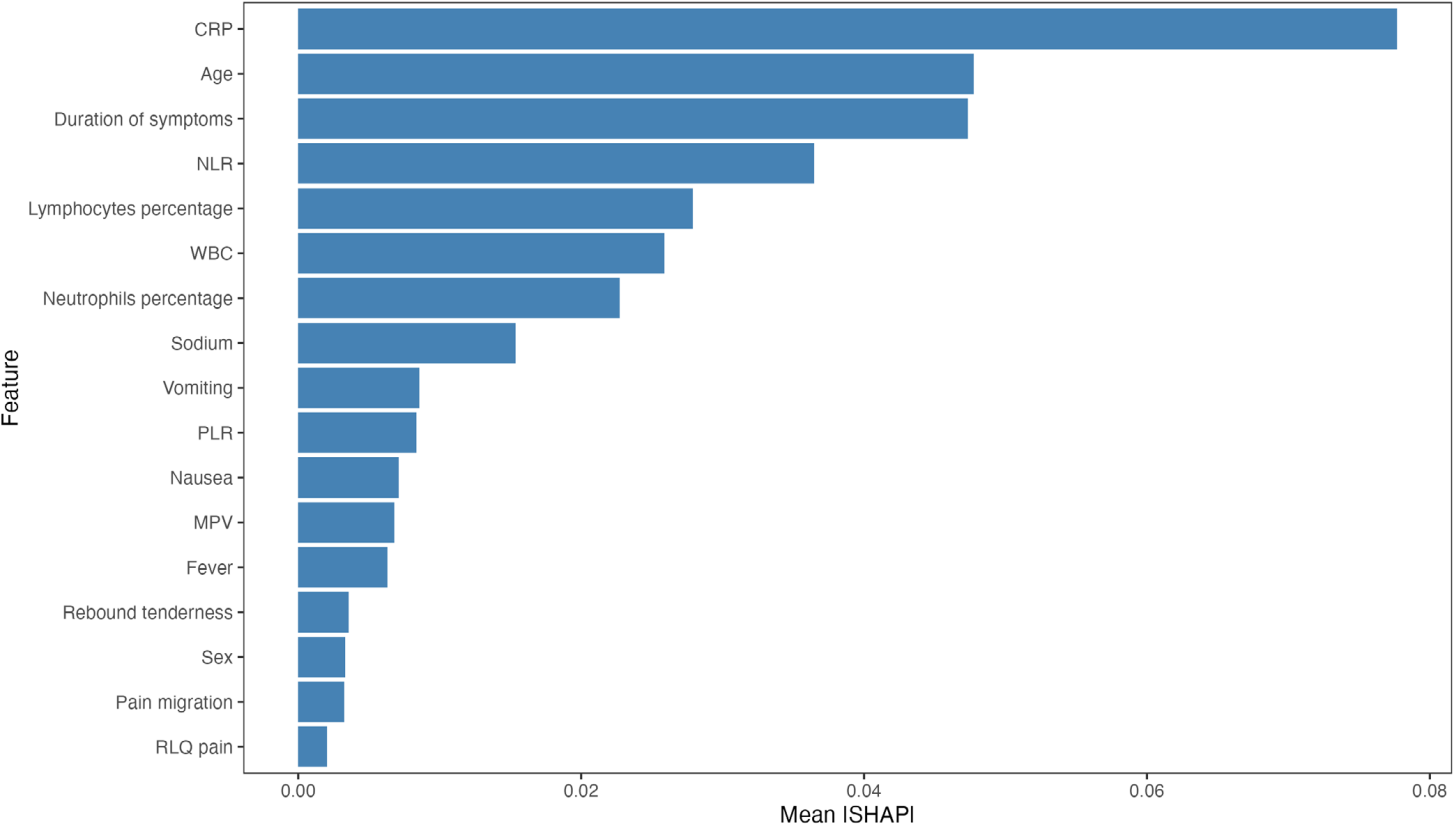
Feature importance of random forest model for complicated appendicitis prediction for the threshold with the highest minimum sensitivity criteria based on Shapley values, shown as average of the absolute value for each fold. Larger values indicate greater overall contribution of that feature to model predictions. LRQ, Lower right quadrant; MPV, Mean platelet volume; NLR, Neutrophil to lymphocyte ratio; PLR, Platelet to lymphocyte ratio.

Shapley values were again calculated with 10 000 permutations for each outer fold and then averaged. For the threshold with the highest minimum sensitivity criteria, the five most important values overall were CRP, age, symptom duration, NLR, and percentage of lymphocytes (Figure 4).

Further SHAP value analysis on the same threshold demonstrated distinct feature importance patterns across prediction outcomes. In true positive predictions, the highest mean absolute SHAP values were observed for CRP, age, symptom duration, NLR, and lymphocyte percentage. False positive predictions were primarily influenced by CRP, symptom duration, age, NLR, and lymphocyte percentage. In true negative predictions, CRP exhibited the greatest SHAP contribution, followed by NLR, lymphocyte percentage, symptom duration, and neutrophil percentage. For false negative predictions, CRP and NLR showed the highest SHAP impact, followed by lymphocyte percentage, neutrophil percentage, and symptom duration.

## 4 Discussion

The persistent negative appendectomy rate of 12%–14% in contemporary practice, despite advanced imaging, represents a significant opportunity for ML‐based decision support [4, 7]. Our study cohort included a total of 623 adult patients with 66 patients (10.6%) with a normal appendix on histology, reflecting this clinical reality. ML models achieving high specificity (while maintaining high sensitivity) could identify very low‐risk patients who might safely avoid surgery through observation or alternative diagnostic approaches.

This study developed and validated four ML models based on clinical and laboratory features. The LR achieved the best performance for acute appendicitis detection with an AUC of 0.765 ± 0.001, while the RF model performed the best for complicated appendicitis prediction with an AUC of 0.785 ± 0.006. Bootstrap validation confirmed model stability, with the LR showing a bootstrap AUC of 0.791 (95% CI 0.713–0.862) for appendicitis detection, while the RF model for complicated appendicitis prediction achieved an AUC of 0.778 (95% CI 0.729–0.828).

It is important to view these results in the context of the targeted population– adult patients who, based on surgeons' judgment, were deemed to have acute appendicitis and consequently received surgical treatment. This is in contrast with studies that developed models with patients who didn't undergo surgery (no histopathological results), but were only clinically and radiologically diagnosed as having acute appendicitis. There were also studies using artificially generated data or general abdominal pain as control groups [8, 9, 12, 13]. Nevertheless, some authors have conducted studies focusing on surgically treated patients with histopathological verification of the diagnosis [14, 15, 16]. Absence of histopathological findings and variable sensitivity and specificity of radiological methods can introduce difficulty in interpreting the results of those studies. Furthermore, patients with general abdominal pain could be missing some of the most important features of acute appendicitis, such as LRQ pain, elevated inflammatory markers, and other related symptoms based on which the surgeon would even make a differential diagnosis of appendicitis [4, 17]. All of the aforementioned factors could introduce a bias towards bigger differences between non‐appendicitis and appendicitis groups used in creating ML models and drive performance up.

Due to the above reasons, only the patients who received histopathological confirmation of appendicitis were considered “true positive” cases, while the patients with normal histopathological finding were “false positives” and there were no “true negatives” (patients with normal appendix and who did not receive surgery) and “false negatives” (appendicitis cases that were missed). Accordingly, specificity and negative predictive value are reported pragmatically and should be interpreted as conditional performance measures within the operated population, reflecting the proportion of patients with histologically normal appendices correctly classified as low risk within a surgically treated cohort rather than diagnostic performance in an unselected emergency department population. From a clinical perspective, the key consideration is not overall discrimination alone, but whether high sensitivity can be maintained while safely identifying a subgroup of patients in whom immediate surgery may be deferred. With the same study design, but in the pediatric population, the RF classifier achieved a sensitivity of 0.997 and specificity of 0.17, outperforming the AIR score [10].

A distinguishing feature of our methodology is the calibration of decision thresholds to achieve predetermined sensitivity targets from 0.95 to 0.995. This approach reflects a target‐prioritization: accuracy in diagnosing true appendicitis cases in order to reduce negative appendectomies. Expressed per 1000 patients using the observed case distribution, utilising the LR model at the highest evaluated sensitivity threshold (minimum sensitivity 0.995) would result in approximately two missed cases of appendicitis and would correctly identify about four patients without appendicitis who could be spared unnecessary surgery. At a lower sensitivity threshold (minimum sensitivity 0.95), the number of missed appendicitis cases would increase to approximately 41 per 1000 patients, while approximately 24 per 1000 patients without appendicitis would be spared surgery. This comparison underscores the trade‐off between diagnostic safety and operative selectivity inherent in threshold‐based model deployment. The observation that LR outperformed other models likely reflects population specific differences in disease presentation. In other words, the better performance of LR suggests that the relationship between inflammatory biomarkers and appendicitis in adults follows patterns amenable to linear modelling, whereas pediatric cohorts may exhibit more complex, non‐linear associations between predictors and outcome. This confirms the need for creation of separate ML models for each population.

The clinical significance of false negative cases depends strongly on how the model would be used in practice. In the present setting, a low‐risk model output should not be interpreted as justification for discharge without further reassessment, but rather as a possible trigger for additional observation, repeat examination, laboratory reassessment, or further imaging before operative intervention. Of note, missed cases commonly were uncomplicated appendicitis. Future analyses should address this directly, as the implications of delayed diagnosis differ substantially between uncomplicated and complicated disease.

Our findings for appendicitis detection with LR achieving AUC of 0.765 compare favourably with recent systematic reviews of ML algorithms in appendicitis diagnosis. A 2023 systematic review examining 29 studies on artificial intelligence in appendicitis found that reported AUROCs ranged broadly across studies, with AUC ranging from 0.677 to 0.87 for LR in terms of appendicitis diagnosis while the studies itself were variable in terms of design and population [11]. A 2024 review by Bhandarkar et al. found that LR was the best performing model in 18% of the studies based on the accuracy alone among all types of diagnostic studies while RF was third with 13%. The AUC values from other studies ranged from 0.619 to 0.976, but were much less commonly reported despite being a better indicator of predictive model performance at multiple thresholds [18]. However, it should be noted that there is no uniformity between studies in terms of number and type of features, and the characteristics of the target population which makes it impossible to directly compare their results with ones in this study.

In terms of predicting the type of appendicitis (uncomplicated vs. complicated), the best performing model was RF, achieving an AUC of 0.785, with maximum achievable sensitivity of 0.997 paired with specificity of 0.057. This capability is clinically valuable as complicated appendicitis typically requires surgical intervention and carries higher morbidity and mortality risks, while for the uncomplicated types some centres recommend trying conservative treatment first [19]. These AUC results align with other ML studies which reported AUC values ranging from 0.574 to 0.996 [9, 20, 21, 22]. Specifically, some of the studies incorporated RF and reported AUC values ranging from 0.75 to 0.996 [9, 20]. For each of those, results should be interpreted and depend on the model, selected population and population size, parameters, inclusion of radiological diagnostic modalities and imbalance adjusting. The model for complicated appendicitis prediction may have value primarily as a stratification tool rather than a definitive classifier. In practice, a high predicted risk of complicated disease could support more urgent operative management, reduce enthusiasm for non‐operative treatment, or prompt additional imaging and closer monitoring. Further subclassification into specific pathological subtypes such as gangrene, perforation, or abscess would have substantially reduced the number of cases in each category, thereby limiting model stability, interpretability, and clinical usefulness in the present dataset.

Additionally, our observed AUC values should be interpreted in relation to the study objective and population. These models were not developed as stand‐alone diagnostic alternatives to CT in an unselected emergency department population, but as threshold‐calibrated decision‐support tools within a surgically enriched cohort. In this setting, even moderate discrimination may still have clinical value if it allows identification of a small subgroup at sufficiently low predicted risk to justify further observation or additional imaging rather than immediate appendectomy.

The integration of explainable AI through SHAP analysis addresses a critical barrier to clinical ML adoption—the “black box” problem. Clinicians require transparent decision processes to appropriately trust and implement algorithmic recommendations [23]. Our SHAP‐based results provide an interpretable summary of feature contributions, enabling clinician understanding of predictions and identification of cases where model recommendations warrant additional clinical judgment or imaging evaluation.

The SHAP‐based feature importance analysis revealed that hematologic inflammatory markers, particularly neutrophil percentage, NLR, and PLR—were the most influential predictors for appendicitis detection and CRP, duration of symptoms, and NLR for complication stratification. This finding aligns with the pathophysiology of acute appendicitis, wherein mucosal bacterial invasion triggers a systemic inflammatory cascade manifesting as leukocytosis, left shift with absolute neutrophilia, and rise of values of CRP as the symptoms progress [24]. Akbulut et al. identified total bilirubin, PNR, PDW, MCW, WBC, and CRP as the most important features of their CatBoost model for appendicitis prediction, while CRP, PDW, AGE, MPV, and total bilirubin were the most important features for prediction of complicated disease [25]. Similarly, Wei et al. reported on total bilirubin, duration of symptoms, temperature, WBC, neutrophils, and NLR as features with the highest SHAP values for their gradient boost model [22]. While Marcinevics et al. used model built‐in measures of importance, they reported appendix diameter on ultrasound, abdominal guarding—presence of peritonitis, WBC count, neutrophil percentage, and CRP as one of the most important predictors [9]. The prominence of PLR in our analysis reflects recent literature recognizing both neutrophil‐driven and platelet‐mediated inflammatory responses in appendicitis severity [26].

These findings should be interpreted in the context of the study population, which consisted exclusively of adult patients already selected for appendectomy based on surgical judgment. Accordingly, the proposed models should not be regarded as primary diagnostic tools for patients presenting with undifferentiated abdominal pain in the emergency department, nor as replacements for established diagnostic pathways or imaging‐based assessment in centres where ultrasound and especially MSCT are routinely available and strongly influence management. Rather, their most plausible role is as decision‐support or reassessment tools within a surgically selected population. Using routinely available clinical and laboratory data, they may help identify a small subgroup of patients in whom immediate surgery could be reconsidered and observation, repeat assessment, or additional imaging considered—especially in settings with limited imaging access or after equivocal imaging findings—when model predictions are discordant with the initial surgical plan. However, such models should be used to prompt reassessment rather than to determine management independently.

Several limitations warrant consideration. First, our retrospective single‐center design limits generalizability to other populations and healthcare systems. Possible regional variations in appendicitis presentation, patient demographics, and clinical practices may affect model performance in different settings. Second, larger datasets and a possibly increased number of features would enable more robust model training and potentially improved performance, particularly for the complicated appendicitis class which comprised 49.6% of the cohort. Also, the percentage of negative appendectomy rate in our cohort may reflect institutional or population‐specific factors and may not generalize to centres with routine preoperative imaging protocols which consequently should have a lower rate of negative findings or to centres which utilize laparoscopy as a method of diagnostics more and have a higher rate of negative findings. All of which leads to the imbalanced dataset due to the population of patients out of which everyone received surgical treatment. However, because of that, we have a histopathology report as an advantage as we're completely sure of final diagnosis. Furthermore, both strength and relative weakness is that the model relies exclusively on clinical and laboratory parameters and excludes imaging findings. We argue that using imaging findings, not in terms of radiomics, may inflate performance metrics of these models as imaging modalities are a diagnostic tool, which improve overall performance as shown by an increase in AUC values from 0.09 to 0.11 depending on the model used [9]. Also, this approach ensures broad applicability even in resource‐limited settings. The present findings should also be interpreted in light of the single centre retrospective design and the institutional context in which the data were generated. The observed performance may have been influenced by local diagnostic pathways, surgeon thresholds for operative intervention, case mix, and patterns of imaging utilization. Finally, the observed performance may have been influenced by local diagnostic pathways, surgeon thresholds for operative intervention, case mix, and patterns of imaging utilization. Preoperative imaging use was not systematically analysed as a study variable in the present study, which limits our ability to determine how local diagnostic strategy affected case selection and model performance. External validation in independent cohorts with different diagnostic workflows and with direct characterization of the false negative subgroup will therefore be necessary before broader clinical application can be considered.

## 5 Conclusion

This study demonstrates that ML models trained on routinely available clinical and laboratory data can provide diagnostic support for acute appendicitis and for the differentiation of complicated from uncomplicated disease in adult surgical patients. By explicitly applying threshold shifting to prioritize high sensitivity, the models were calibrated to reflect clinically relevant decision‐making contexts in which avoiding missed appendicitis is paramount in order to reduce the number of negative appendectomies. Model robustness was supported through nested cross‐validation and complementary bootstrap analyses, which consistently demonstrated stable performance across resampling. Furthermore, the use of SHAP‐based explainability allowed transparent interpretation of model behaviour, confirming that inflammatory markers such as CRP, WBC, neutrophil percentage, and derived ratios were the primary drivers of predictions, in line with established pathophysiological understanding.

Despite these strengths, the clinical applicability of the proposed models remains constrained by the retrospective, single‐centre design and by the restriction to surgically treated patients. Consequently, these models should be regarded as adjunctive decision‐support tools within a surgically selected adult population rather than standalone diagnostic instruments for general emergency department use. Prospective external and multicentric validation with same study design will be essential to determine their true impact on clinical workflows and outcomes, especially considering possible different diagnostic workflows in different institutions. Future research should explore integration of clinical, laboratory, and imaging data into unified multimodal ML models leveraging deep learning architectures capable of processing heterogeneous information types. Future studies may explore whether ML–based decision support could assist in the selection of patients with uncomplicated appendicitis for non‐operative management, in the context of centres using this treatment strategies. Development of models predicting specific complications (perforation, abscess formation) or optimal surgical timing could further refine clinical decision‐making.

## Author Contributions

**Ivan Males:** conceptualization, methodology, data collection, investigation, formal analysis, writing – original draft. **Marko Kumric:** investigation, formal analysis, writing – review and editing. **Zvonimir Boban:** investigation, formal analysis, writing – review and editing. **Josip Vrdoljak:** visualization, investigation, formal analysis, writing – review and editing. **Dario Pecenkovic:** data curation, formal analysis, writing – review and editing. **Matej Ivanda:** data curation, formal analysis, writing – original draft. **Marko Grahovac:** data curation, investigation, writing – original draft. **Zenon Pogorelic:** writing – review and editing, supervision, investigation. **Josko Bozic:** conceptualization, resources, project administration, writing – review and editing.

## Funding

The authors have nothing to report.

## Ethics Statement

The study was approved by the Ethics Committee of the University Hospital of Split (Approval number: 500–03/23–01/169; approval date: July 21, 2023) and conducted in accordance with the ethical principles of the Declaration of Helsinki. All personal data were anonymized prior to analysis.

## Conflicts of Interest

The authors declare no conflicts of interest.

## Data Availability

The data that support the findings of this study are available from the corresponding author upon reasonable request.

## Appendix

**TABLE A1:** Bootstrap analysis of the developed models for appendicitis detection.

Model and thresholds	AUC	Sensitivity	Specificity	Accuracy	PPV	NPV	F1
LR							
Threshold 1	0.791 (0.713–0.862)	0.936	0.307	0.868	0.919	0.378	0.927
		(0.878–0.984)	(0.12–0.524)	(0.826–0.905)	(0.882–0.954)	(0.182–0.636)	(0.902–0.948)
Threshold 2		0.946	0.273	0.874	0.916	0.391	0.930
		(0.892–0.986)	(0.099–0.5)	(0.834–0.908)	(0.878–0.953)	(0.188–0.667)	(0.907–0.951)
Threshold 3		0.969	0.179	0.885	0.908	0.427	0.937
		(0.93–0.995)	(0.038–0.363)	(0.851–0.916)	(0.872–0.943)	(0.143–0.799)	(0.918–0.955)
Threshold 4		0.975	0.152	0.887	0.906	0.441	0.939
		(0.941–1)	(0.032–0.315)	(0.853–0.918)	(0.87–0.942)	(0.125–1)	(0.919–0.957)
Threshold 5		0.986	0.098	0.890	0.901	0.473	0.942
		(0.959–1)	(0–0.234)	(0.858–0.922)	(0.867–0.935)	(0–1)	(0.923–0.959)
Threshold 6		0.993	0.053	0.893	0.898	0.532	0.943
		(0.976–1)	(0–0.167)	(0.863–0.924)	(0.865–0.93)	(0–1)	(0.926–0.961)
RF							
Threshold 1	0.759 (0.677–0.841)	0.901	0.403	0.847	0.927	0.332	0.913
		(0.832–0.955)	(0.222–0.625)	(0.795–0.888)	(0.891–0.962)	(0.186–0.5)	(0.88–0.937)
Threshold 2		0.925	0.338	0.862	0.921	0.356	0.923
		(0.872–0.969)	(0.16–0.542)	(0.821–0.898)	(0.885–0.962)	(0.185–0.556)	(0.898–0.944)
Threshold 3		0.945	0.271	0.873	0.915	0.377	0.93
		(0.904–0.98)	(0.115–0.455)	(0.838–0.905)	(0.879–0.949)	(0.188–0.611)	(0.909–0.948)
Threshold 4		0.956	0.229	0.912	0.912	0.388	0.933
		(0.92–0.986)	(0.08–0.409)	(0.877–0.946)	(0.877–0.946)	(0.182–0.636)	(0.914–0.951)
Threshold 5		0.971	0.154	0.884	0.906	0.408	0.937
		(0.939–0.995)	(0.036–0.3)	(0.851–0.917)	(0.871–0.939)	(0.143–0.799)	(0.918–0.956)
Threshold 6		0.98	0.109	0.887	0.902	0.427	0.939
		(0.949–1)	(0–0.25)	(0.855–0.919)	(0.869–0.935)	(0–1)	(0.921–0.957)
BRF							
Threshold 1	0.759 (0.677–0.839)	0.879	0.455	0.833	0.931	0.312	0.904
		(0.819–0.932)	(0.273–0.647)	(0.783–0.878)	(0.899–0.962)	(0.184–0.462)	(0.871–0.931)
Threshold 2		0.912	0.377	0.855	0.925	0.341	0.918
		(0.861–0.956)	(0.208–0.563)	(0.813–0.894)	(0.893–0.956)	(0.2–0.5)	(0.892–0.942)
Threshold 3		0.938	0.301	0.869	0.918	0.369	0.928
		(0.898–0.971)	(0.148–0.471)	(0.831–0.945)	(0.885–0.949)	(0.192–0.563)	(0.904–0.948)
Threshold 4		0.943	0.283	0.873	0.917	0.376	0.93
		(0.904–0.975)	(0.136–0.45)	(0.835–0.907)	(0.884–0.948)	(0.188–0.583)	(0.907–0.95)
Threshold 5		0.966	0.185	0.883	0.909	0.402	0.936
		(0.935–0.99)	(0.05–0.333)	(0.849–0.917)	(0.876–0.941)	(0.154–0.714)	(0.916–0.956)
Threshold 6		0.974	0.144	0.885	0.905	0.415	0.938
		(0.944–0.995)	(0.038–0.276)	(0.851–0.919)	(0.873–0.936)	(0.143–0.799)	(0.918–0.958)
XGB							
Threshold 1	0.762 (0.674–0.84)	0.914	0.367	0.855	0.924	0.344	0.918
		(0.854–0.964)	(0.192–0.859)	(0.812–0.894)	(0.887–0.958)	(0.188–0.55)	(0.892–0.941)
Threshold 2		0.924	0.339	0.862	0.921	0.357	0.923
		(0.868–0.971)	(0.172–0.542)	(0.819–0.899)	(0.886–0.955)	(0.194–0.583)	(0.897–0.945)
Threshold 3		0.948	0.269	0.875	0.916	0.389	0.931
		(0.9–0.985)	(0.107–0.458)	(0.835–0.909)	(0.881–0.949)	(0.188–0.643)	(0.908–0.951)
Threshold 4		0.954	0.245	0.878	0.914	0.402	0.933
		(0.911–0.986)	(0.094–0.428)	(0.84–0.912)	(0.879–0.948)	(0.182–0.667)	(0.911–0.953)
Threshold 5		0.971	0.178	0.886	0.908	0.439	0.938
		(0.936–0.995)	(0.042–0.348)	(0.849–0.919)	(0.873–0.942)	(0.166–0.799)	(0.917–0.957)
Threshold 6		0.976	0.154	0.889	0.906	0.457	0.94
		(0.944–0.996)	(0.034–0.308)	(0.855–0.92)	(0.872–0.939)	(0.143–0.857)	(0.92–0.958)

*Note:* Thresholds 1–6 – thresholds with minimum sensitivity of 0.95, 0.96, 0.97, 0.98, 0.99, and 0.995. Values are presented as mean (95% CI).

Abbreviations: AUC, area under the curve; BRF, balanced random forest; F1, F1 score; LR, logistic regression; NPV, negative predictive value; PPV, positive predictive value; RF, random forest; XGB, eXtreme Gradient Boosting.

**TABLE A2:** Bootstrap analysis of the developed models for complicated appendicitis prediction.

Model and thresholds	AUC	Sensitivity	Specificity	Accuracy	PPV	NPV	F1
LR							
Threshold 1	0.778 (0.729–0.828)	0.936	0.296	0.611	0.568	0.837	0.705
		(0.874–1)	(0.111–0.477)	(0.516–0.629)	(0.482–0.660)	(0.714–1)	(0.644–0.762)
Threshold 2		0.952	0.238	0.590	0.552	0.848	0.697
		(0.878–1)	(0.076–0.414)	(0.499–0.678)	(0.473–0.639)	(0.721–1)	(0.936–0.755)
Threshold 3		0.961	0.201	0.576	0.542	0.856	0.692
		(0.896–1)	(0.059–0.379)	(0.489–0.664)	(0.469–0.626)	(0.725–1)	(0.634–0.749)
Threshold 4		0.974	0.142	0.554	0.528	0.872	0.684
		(0.919–1)	(0.033–0.302)	(0.479–0.627)	(0.463–0.603)	(0.714–1)	(0.630–0.739)
Threshold 5		0.978	0.127	0.548	0.524	0.879	0.681
		(0.929–1)	(0.025–0.273)	(0.475–0.627)	(0.461–0.595)	(0.714–1)	(0.628–0.736)
Threshold 6		0.985	0.098	0.536	0.517	0.893	0.677
		(0.944–1)	(0.015–0.212)	(0.467–0.609)	(0.457–0.584)	(0.696–1)	(0.626–0.730)
RF							
Threshold 1	0.774 (0.726–0.819)	0.791	0.591	0.689	0.665	0.743	0.717
		(0.696–0.876)	(0.481–0.694)	(0.623–0.737)	(0.576–0.731)	(0.652–0.831)	(0.661–0.764)
Threshold 2		0.798	0.581	0.687	0.651	0.747	0.716
		(0.704–0.882)	(0.471–0.684)	(0.631–0.735)	(0.572–0.728)	(0.656–0.835)	(0.661–0.764)
Threshold 3		0.812	0.559	0.684	0.644	0.754	0.717
		(0.721–0.893)	(0.449–0.667)	(0.628–0.733)	(0.568–0.721)	(0.663–0.845)	(0.664–0.765)
Threshold 4		0.824	0.541	0.680	0.638	0.760	0.718
		(0.739–0.901)	(0.431–0.648)	(0.624–0.731)	(0.562–0.715)	(0.667–0.853)	(0.664–0.766)
Threshold 5		0.867	0.465	0.663	0.614	0.783	0.718
		(0.788–0.938)	(0.358–0.579)	(0.606–0.716)	(0.542–0.688)	(0.686–0.877)	(0.667–0.767)
Threshold 6		0.923	0.329	0.621	0.574	0.816	0.707
		(0.862–0.973)	(0.226–0.445)	(0.557–0.685)	(0.505–0.646)	(0.712–0.917)	(0.655–0.756)
BRF							
Threshold 1	0.768 (0.719–0.816)	0.641	0.738	0.689	0.706	0.677	0.670
		(0.531–0.736)	(0.645–0.819)	(0.638–0.742)	(0.624–0.787)	(0.601–0.754)	(0.605–0.733)
Threshold 2		0.687	0.692	0.689	0.687	0.693	0.685
		(0.589–0.782)	(0.596–0.782)	(0.637–0.741)	(0.607–0.761)	(0.613–0.774)	(0.623–0.744)
Threshold 3		0.698	0.680	0.689	0.682	0.698	0.689
		(0.599–0.794)	(0.585–0.771)	(0.635–0.741)	(0.603–0.756)	(0.615–0.779)	(0.624–0.747)
Threshold 4		0.718	0.659	0.687	0.675	0.705	0.694
		(0.621–0.810)	(0.557–0.752)	(0.631–0.740)	(0.597–0.750)	(0.622–0.786)	(0.632–0.751)
Threshold 5		0.862	0.484	0.671	0.621	0.784	0.721
		(0.776–0.934)	(0.385–0.580)	(0.617–0.720)	(0.556–0.689)	(0.684–0.881)	(0.669–0.770)
Threshold 6		0.909	0.395	0.649	0.596	0.819	0.719
		(0.839–0.965)	(0.298–0.495)	(0.593–0.704)	(0.532–0.663)	(0.714–0.917)	(0.669–0.768)
XGB							
Threshold 1	0.779 (0.733–0.826)	0.907	0.368	0.634	0.585	0.804	0.710
		(0.837–0.964)	(0.252–0.486)	(0.571–0.694)	(0.518–0.658)	(0.702–0.905)	(0.660–0.761)
Threshold 2		0.921	0.334	0.624	0.576	0.814	0.707
		(0.863–0.973)	(0.225–0.454)	(0.563–0.686)	(0.508–0.648)	(0.705–0.923)	(0.657–0.758)
Threshold 3		0.939	0.282	0.607	0.562	0.831	0.703
		(0.879–0.989)	(0.175–0.398)	(0.541–0.672)	(0.495–0.634)	(0.717–0.955)	(0.652–0.755)
Threshold 4		0.952	0.243	0.594	0.553	0.845	0.699
		(0.898–0.991)	(0.142–0.356)	(0.526–0.659)	(0.488–0.622)	(0.724–0.964)	(0.646–0.749)
Threshold 5		0.966	0.199	0.578	0.542	0.864	0.694
		(0.919–1)	(0.103–0.311)	(0.513–0.645)	(0.478–0.609)	(0.733–0.1)	(0.642–0.745)
Threshold 6		0.968	0.193	0.576	0.541	0.867	0.693
		(0.920–1)	(0.096–0.301)	(0.509–0.643)	(0.476–0.608)	(0.732–1)	(0.641–0.743)

*Note:* Thresholds 1–6 – thresholds with minimum sensitivity of 0.95, 0.96, 0.97, 0.98, 0.99, and 0.995. Values are presented as mean (95% CI).

Abbreviations: AUC, area under the curve; BRF, balanced random forest; F1, F1 score; LR, logistic regression; NPV, negative predictive value; PPV, positive predictive value; RF, random forest; XGB, eXtreme Gradient Boosting.
